# Intercalation and Retention of Carbon Dioxide in a Smectite Clay promoted by Interlayer Cations

**DOI:** 10.1038/srep08775

**Published:** 2015-03-05

**Authors:** L. Michels, J. O. Fossum, Z. Rozynek, H. Hemmen, K. Rustenberg, P. A. Sobas, G. N. Kalantzopoulos, K. D. Knudsen, M. Janek, T. S. Plivelic, G. J. da Silva

**Affiliations:** 1 Department of Physics, Norwegian University of Science and Technology, NTNU. Trondheim, Norway; 2 Physics Department, Institute for Energy Technology, IFE, Kjeller, Norway; 3 Slovak University of Technology, Bratislava, Slovakia; 4 MAX IV Laboratory, Lund University, Lund, Sweden; 5 Instituto de Física and International Center for Condensed Matter Physics, Universidade de Brasília, Brasília, Brasil

## Abstract

A good material for CO_2_ capture should possess some specific properties: (i) a large effective surface area with good adsorption capacity, (ii) selectivity for CO_2_, (iii) regeneration capacity with minimum energy input, allowing reutilization of the material for CO_2_ adsorption, and (iv) low cost and high environmental friendliness. Smectite clays are layered nanoporous materials that may be good candidates in this context. Here we report experiments which show that gaseous CO_2_ intercalates into the interlayer nano-space of smectite clay (synthetic fluorohectorite) at conditions close to ambient. The rate of intercalation, as well as the retention ability of CO_2_ was found to be strongly dependent on the type of the interlayer cation, which in the present case is Li^+^, Na^+^ or Ni^2+^. Interestingly, we observe that the smectite Li-fluorohectorite is able to retain CO_2_ up to a temperature of 35°C at ambient pressure, and that the captured CO_2_ can be released by heating above this temperature. Our estimates indicate that smectite clays, even with the standard cations analyzed here, can capture an amount of CO_2_ comparable to other materials studied in this context.

Interactions between CO_2_ and clay minerals have attracted interest in the scientific community in recent years, partly because geological structures are being investigated as storage sites for anthropogenic CO_2_. The cap-rock formations which act as flow barriers and seals in this context are known to contain high proportions of clay minerals^1^, and the long-term integrity of these formations is a prerequisite for avoiding CO_2_ losses to the atmosphere^2^,^3^. However, the physical parameters affecting the interactions between CO_2_ and clay minerals under reservoir conditions are still not well understood^4^.

Clay minerals are materials based on two-dimensional stacks of inorganic layers^5^. In some clay minerals (smectites), non-equivalent substitutions of atoms generate a negative charge on each layer surface which is balanced by exchangeable interlayer cations. These cations are responsible for the differences in the physico-chemical behavior of smectites such as water adsorption and retention, plasticity, swelling etc^6^,^7^. Smectite clay mineral particles typically consist of approximately hundred layers. Smectites have the ability to intercalate additional molecules into the interlayer space, thereby changing the repetition distance along the layer normal (z-direction), a process which is known as swelling^8^,^9^. Intercalation of water can also occur, since H_2_O is a polar molecule, and this has been extensively studied with a wide range of techniques, such as neutron^8^,^10^,^11^ and X-ray scattering^9^,^12^,^13^, NMR spectroscopy^14^,^15^,^16^ tracer experiments^17^ or numerical modeling^14^,^18^.

Experiments^19^,^20^,^21^,^22^,^23^,^24^,^25^,^26^,^27^,^28^ and simulations^18^,^29^,^30^,^31^,^32^ have also shown that CO_2_ intercalates in some smectite clays, both in supercritical and in gaseous/liquid form. We have recently demonstrated that CO_2_ is able to intercalate in Na-fluorohectorite (NaFh) smectite clay mineral at conditions close to ambient (−20°C, 5 bar)^22^. In that work we also showed that under the same conditions neither H_2_O vapor nor N_2_ gas intercalates. These are not the typical conditions found in geological storage sites, but the conditions are relevant if clays are considered as a potential material for the capture or sequestration of CO_2_, and it is also of interest to study CO_2_ capture and retention under these conditions for the purpose of understanding the underlying molecular mechanisms. Several porous materials are currently being assessed for the purpose of CO_2_ capture and retention^33^,^34^,^35^. In this context, clay-containing materials could have a distinct advantage in that they are both cheap and ubiquitous^31^, and also because they generally provide a very large accessible effective surface area that arises from nanolayered stacked structures embedded in a mesoporous powder matrix.

Fluorohectorites (Fh) are synthetic smectites which have been used as a representative and clean model system of natural smectite clays^36^. Synthetic clays have the advantage that they possess a more homogeneous charge distribution, and also contain significantly fewer impurities (e.g. carbonates, (hydr)oxides, silica, and organic matter) than their natural counterparts^7^.

In the present work we studied the intercalation of CO_2_ in Li-fluorohectorite (LiFh), Na-fluorohectorite (NaFh) and Ni-fluorohectorite (NiFh). The only differences between these samples are the interlayer charge compensating cations used. Intercalation experiments under different temperature and pressure conditions were conducted. In order to investigate the potential of fluorohectorite clays for CO_2_ storage and capture, we also quantified the CO_2_ adsorption (wt%).

## Results

### CO_2_ Intercalation

**Figure 1:**
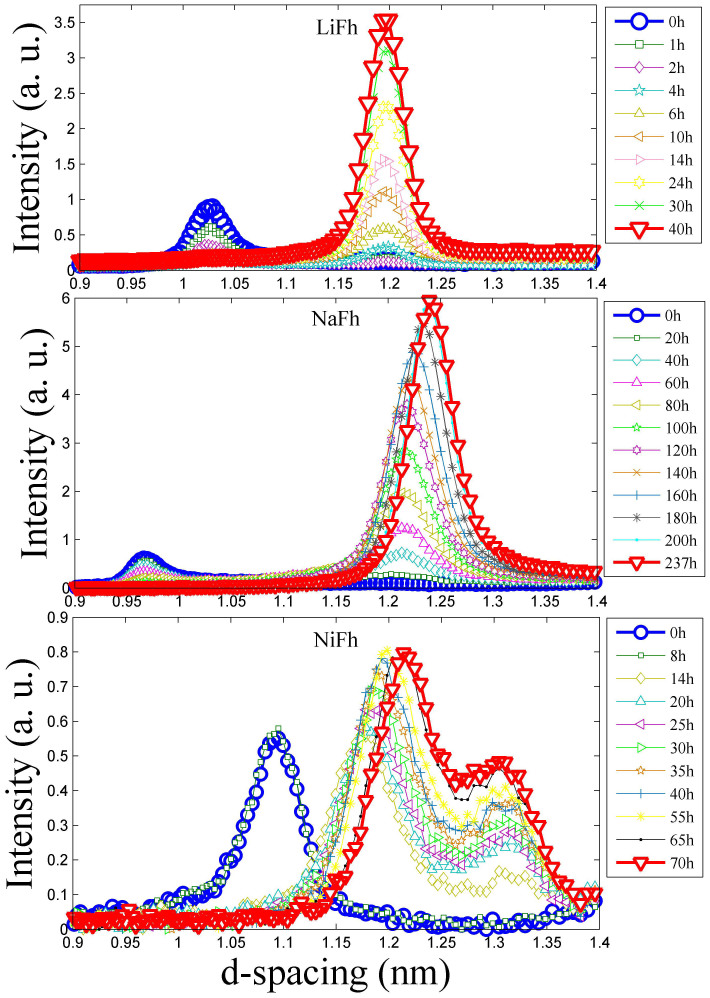
The scattering intensity (arb. units) over a range of *d-spacing* values at different times (hours), for LiFh, NaFh and NiFh, respectively. In all cases the CO_2_ adsorption conditions were −20°C and 20 bar. The red line (∇) shows the time at saturation. Experiment performed at NTNU.

The layered nature of smectite clays gives rise to well-defined (00l) diffraction peaks, and the angular position of these peaks is a direct measure of the interlayer repetition distance (the *d-spacing*). Figure 1 displays how the intensity of the (001) diffraction peak grows with time as the samples are exposed to CO_2_ at −20°C and 20 bar. All the samples were pre-dried before the measurements (see Methods section below). Intercalation of CO_2_ in the interlayer space manifests itself as the growth of an intercalation peak at a lower scattering angle (higher *d-spacing*) than the peak of dry, non-intercalated clay. As the intercalation progresses, the intensity of the CO_2_-intercalation peaks increase whereas the scattering from non-intercalated part of the sample decreases and eventually vanishes. LiFh and NaFh show similar intercalation behaviors in the X-ray diffractogram (XRD). The (001) peaks develop to d-spacings of 1.196 nm and 1.240 nm, for LiFh and NaFh respectively. This could correspond to a monolayer of intercalated CO_2_, in analogy to what occurs for H_2_O. For the NiFh sample we observe a similar intercalation state with d-spacing of 1.219 nm, and in addition the development of another state with a larger d-spacing of 1.311 nm. To our knowledge, this is the first time such a complex CO_2_ intercalation state has been observed in a clay mineral, although other authors have found evidence of multiple intercalated layer type^9^,^37^. One may note that the secondary low-angle peak is at a *d-spacing* of ca. 1.3 nm, which is distinct from the ≈1.25 and ≈1.55 nm spacings of the one (1WL) and two water layer (2WL) smectite states as reported by Ferrage et al^38^,^39^. Other XRD studies have generally observed only blurred peaks in this region and have interpreted them as mixtures of peaks with the 1WL and 2WL spacings.

**Figure 2:**
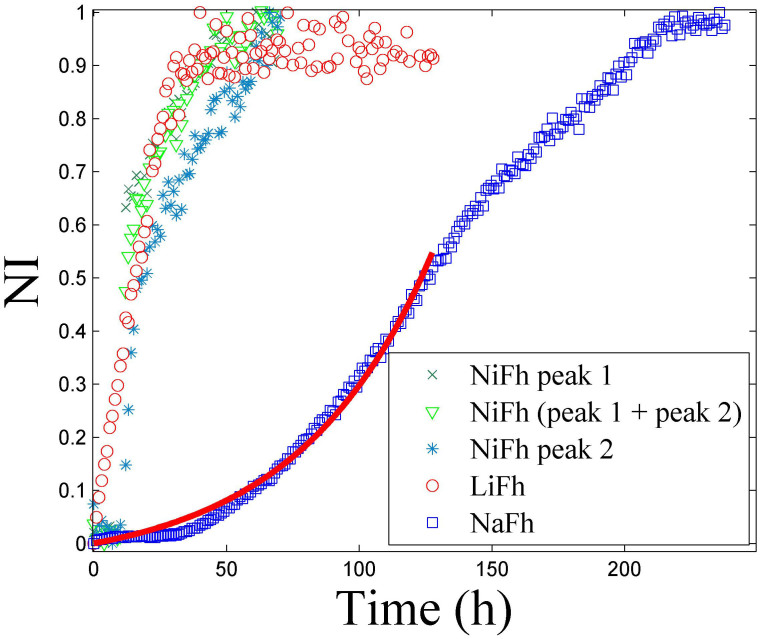
Intensity of the intercalation peak, normalized to the maximum intensity (NI), as a function of time for different cations at 20 bar and −20°C. The NiFh diffractogram has two contributions: Peak 1 is the peak with the maximum intensity with *d-spacing* ≈ 1.21 nm while peak 2 is the peak with *d-spacing* ≈ 1.31 nm.

Figure 2 shows the comparison of (001) peak intensity vs. time for LiFh, NaFh and NiFh. In this Figure we plot the NiFh (001) peak intensities of *d-spacing* ≈ 1.21 nm, *d-spacing* ≈ 1.31 nm and the sum of them. We observe that the intercalation rate is significantly higher for NiFh and LiFh than for NaFh. This is similar to the case of water intercalation, where cations have been found to determine the stable states at varying relative humidity^18^,^40^,^41^,^42^,^43^ as well as the way that clay minerals exfoliate in aqueous dispersion^6^,^44^. Fripiat et al.^27^ suggested that the access of CO_2_ molecules to the interlayer space of montmorillonite clay is dependent on the size of the interlayer cation. Giesting et al.^23^ studied CO_2_-intercalation behavior of K-and Ca-montmorillonite, performing repeated measurements under the same conditions, and also reported a significant dependence of dynamics on the cations. The dynamics of the CO_2_ intercalation can also be followed by observing the disappearance of the scattering intensity of the dehydrated peak, shown in Figure S1 in supporting information, which represents the d-spacing in the portion of the sample with no water or CO_2_ intercalated. In Figure S2 of the supporting information we show the d-spacing dynamics of the CO_2_ intercalated peak for each sample. Although the procedure used involves pre-drying of the samples it is possible that there is a minor amount of remaining H_2_O within the sample. This amount must in any case be very small since there is no detectable 1WL peak in the XRD patterns. Any residual H_2_O could affect the kinetics of the CO_2_ intercalation.

**Figure 3:**
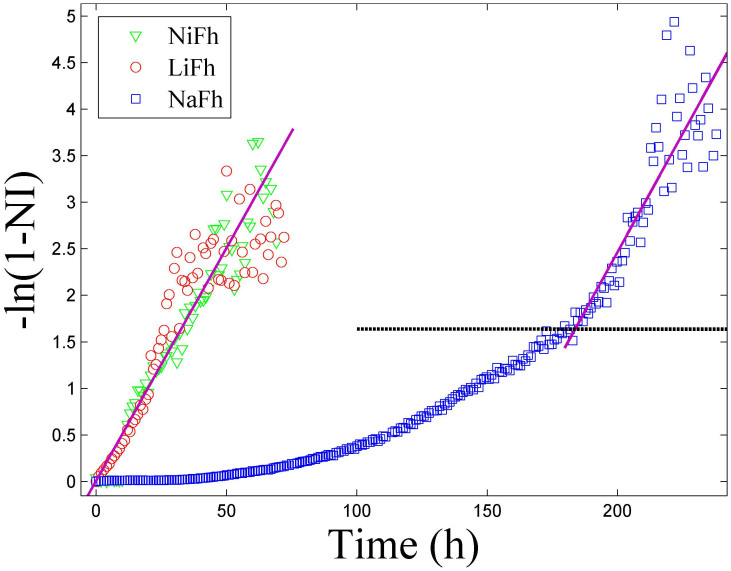
The linearized intensity as function of time. The horizontal line represents the threshold between two regimes for NaFh: Clay swelling accompanied by some intercalation of CO_2_ (below the horizontal line), which is described by the exponential function 
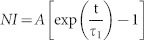
, and intercalation of CO_2_ into the swollen interlayer of the clay mineral (above the horizontal line) described by equations (2) and (3). The initial swelling part of the dynamics is much faster for NiFh and LiFh as compared to NaFh, which is also confirmed in Figure S1 in the supporting information.

A general equation for describing sorption kinetics is^45^:
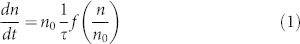
where n is the amount of adsorbed molecules on a surface and 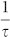 is a rate constant that depends on temperature and pressure. The equation for the function 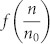 depends on the type of adsorption mechanism. For a first order adsorption process, i.e. a process where the adsorbed molecules statistically occupy a single adsorption site^46^, 
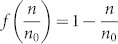
. In the present case, 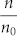 is proportional to the normalized X-ray intensity (*NI*), where normalization is performed with respect to the intensity observed at the longest times, where the adsorption capacity of the material is reached. By integrating equation (1), we obtain
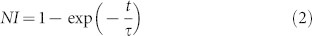
which is an exponential growth function towards saturation at 1. Applying the natural logarithm, we have a linear equation with the slope proportional to the rate constant 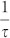:
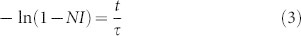


The results of plotting the observed normalized intensity in this manner are shown in Figure 3. It is evident that equation (3) describes the data for NiFh and LiFh reasonably well, before the full adsorption capacity is reached, i.e. before all the sites have been occupied. However, for NaFh we observe deviation for −ln(1 − *NI*) < 1.6, which we relate to the Na^+^ ion providing a stronger layer adherence than the other two ions investigated here, and thus the swelling is more difficult to achieve for the case of Na^+^, since the clay layers are closer together in the dehydrated state of NaFh, as shown in Figure 1 (*d-spacing* ≈ 0.97 nm). The difference in *d-spacing* between the dehydrated and the CO_2_ intercalated peaks is almost two times higher for NaFh compared to LiFh and NiFh (Figure 1). The horizontal line in Figure 3 represents the threshold between two regimes for the case of NaFh: Clay expansion accompanied by partial adsorption of CO_2_ (below the line), and adsorption of CO_2_ into the expanded interlayer of the clay mineral (above the line). This is in agreement with Figure 1, where it is noticeable that for the case of NaFh there is small shift with time of the monolayer CO_2_ Bragg peak even after expansion, indicating that more CO_2_ is adsorbed into the interlayer.

After the swelling process is finished, which means that the clay does not expand significantly anymore, the intercalation process is cation independent since the slopes of the curves shown in Figure 3 are the same for all samples, i.e. 
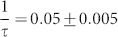
 hours^−1^. This corresponds to a time constant *τ* of approx. 20 hours. A single exponential growth function 
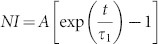
 (shown in Figure 2), with τ_1_ ≈ 51 hours, related to the clay swelling, describes well the data in the initial phase for NaFh, i.e. for −ln(1 − *NI*) < 1.6 in Figure 3.

**Figure 4:**
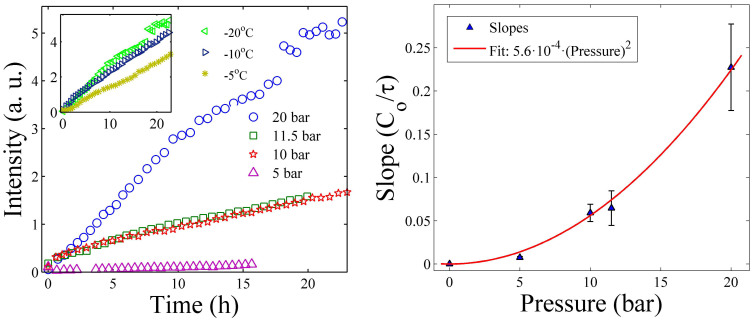
Left panel: The intensity of the (001) CO_2_ intercalation peak in LiFh as a function of time for different pressures (at constant temperature of −20°C). The intercalation rate increases with the pressure. Right panel: The quadratic dependence of the linear fit slopes *C_o_/τ* on CO_2_ pressure. Experiment performed at Maxlab.

The dependence of the intercalation intensity at various pressures is shown in Figure 4 for LiFh. The results show that the intercalation rate increases with pressure and this observation is in agreement with a previous study of NaFh^22^. We also measured intercalation at various temperatures (at a constant pressure of 20 bar) in the limited temperature range −5, −10 and −20°C and the data suggests (inset of Figure 4) faster adsorption at lower temperature. We have not measured the T-dependence of τ here, but for simplicity, we may assume this to follow an inverse Arrhenius behavior^47^.

The intensity of the CO_2_ intercalation peak follows a linear behavior for small times, in agreement with equation (2), which for short times *t* ≪ *τ* becomes *NI* ≈ *t*/*τ*. In Figure 4 we have not normalized the measured intensities to their saturation at long times, because we did not follow the experiments until saturation, thus the slopes (*S*) of the straight lines at short times in Figure 4 (left panel) can be expressed as 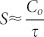 where *C_o_* is a constant. As suggested above, one can assume that the adsorption dynamics after swelling is governed by an average inverse Arrhenius like trapping time:
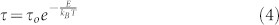
where, 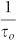 is an average attempt frequency, *E* is an average energy barrier, *k_B_* is Boltzmann constant and *T* is the absolute temperature. Further we can assume that the attempt frequency 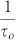 increases with pressure, *P*, i.e. the higher the pressure, the more attempts are made by the CO_2_ molecules to cross the adsorption trapping energy barrier *E*. In the right panel of Figure 4, we have tested this assumption and fitted an empiric parabolic function to the pressure dependence of 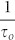. Our temperature measurements are in such a limited range in absolute temperature that they do not allow us to test inverse Arrhenius trapping time assumption or any other temperature model for τ, but the desorption data for LiFh (shown below) indicates that the sorption trapping barrier is of the order of magnitude of 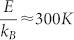. This gives 
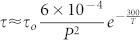
 where *P* is in units of bar and *T* in units of absolute temperature (K). From Figure 3 we estimated *τ* ≈ 20 hours for *P* and *T* equal to 20 bar and 253 K, respectively. This enables us to estimate *τ_o_* ≈ 5 · 10^7^ hours, and thus the adsorption time would be *τ* ≈ 14 min for *P* and *T* equal to 200 bar and 250 K respectively, if we assume that trapping mechanism for liquid and gas CO_2_ are the same.

### CO_2_ retention under ambient conditions

**Figure 5:**
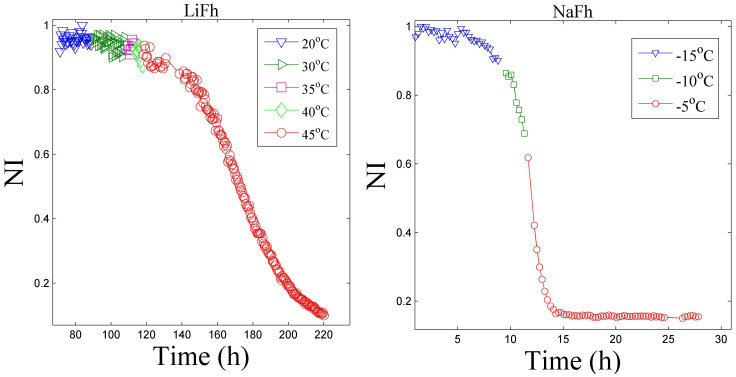
Normalized Intensity (NI) of the (001) peak of LiFh (left) and NaFh (right) as a function of time for increasing temperatures. Experiments performed at NTNU.

After exposing the three types of clay mineral samples to CO_2_ pressure for a sufficiently long time, the CO_2_ pressure was released and the cell was continuously flushed with N_2_, at atmospheric pressure, while increasing the temperature in steps of 5°C. It is known that the dry N_2_ does not intercalate into Na-fluorohectorite^22^. A plot of peak intensity versus time at different increasing temperatures is shown in Figure 5.

**Figure 6:**
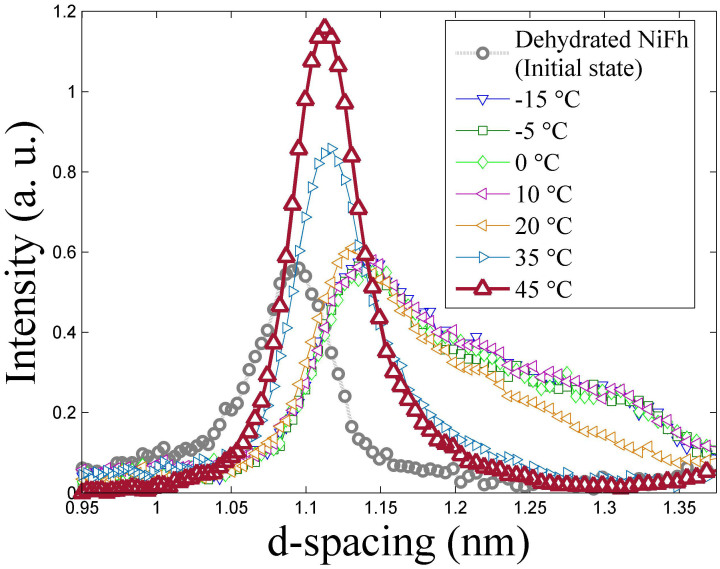
(001) peak intensity of NiFh as a function of the *d-spacing* for different temperatures in N_2_ at atmospheric pressure. As a reference the initial dehydrated peak of NiFh is included in the graph (circles, which is the same as the 0h curve in Figure 1). Experiments performed at NTNU.

It has previously been concluded that the interlayer CO_2_ may cause an irreversible adsorption in clay, i.e. even if a clay sample is not exposed to the CO_2_ gas, CO_2_ molecules remain in the interlayer space^48^,^49^. This means that once intercalated with CO_2_ the clay mineral will retain these molecules. However, a temperature change can affect the CO_2_ retention^50^,^51^ and this makes the process of intercalation and release truly reversible. We found that at a certain threshold temperature, the intensity decreases until the contribution to the scattered intensity from the clay mineral with intercalated CO_2_ is negligible. Simultaneously, the peak corresponding to the dehydrated LiFh and NaFh reappears (data not shown). The threshold temperature, at which the CO_2_ is desorbed from the interlayer space of the clays, is highly dependent on the type of interlayer cation used. For LiFh, this temperature is about 35°C, whereas for NaFh it is about −15°C (Figure 5). This is consistent with the difference in size between the smaller Li^+^ cation versus the larger Na^+^ cation. Li^+^ has a more concentrated charge distribution than Na^+^ and can thus polarize the CO_2_ molecule more, forming a stronger bond to it. Loring et al also give a description of the CO_2_ intercalation mechanism^20^. In the case of NiFh the release, like the intercalation, has more complex features, as shown in Figure 6.

Comparing the NiFh spectrum in Figure 1 with Figure 6 one can see that upon heating, the second CO_2_ peak merges with the first CO_2_ peak for NiFh. With increasing temperature, the intensity of the peak at the highest *d-spacing* value (about 1.31 nm) decreases, and at 45°C it completely disappears while the lowest *d-spacing* value peak shifts to lower values and eventually contains all the (001) scattering. It appears that the final intercalation state is different from the original dehydrated state. This could suggest the formation of a complex CO_2_-Ni^2+^ structure within the interlayer space of the NiFh clay mineral, not present in the case of LiFh and NaFh. It is known that water intercalation experiments with NiFh can form a structure called Brucite (Ni[OH]_2_). Such a structure is formed in the cation exchange process from LiFh to NiFh^60^. It is possible that a Brucite-CO_2_ interaction could have an effect on the behavior. In addition this could occur due to the partially occupied d-orbitals of the Ni^2+^ ions, which allow multiple coordination geometries with CO_2_. These geometries can be possibly achieved by interactions of Ni d-orbitals with free oxygen orbitals present in polarized CO_2_ molecules.

### Pressure composition Temperature Experiments

**Figure 7:**
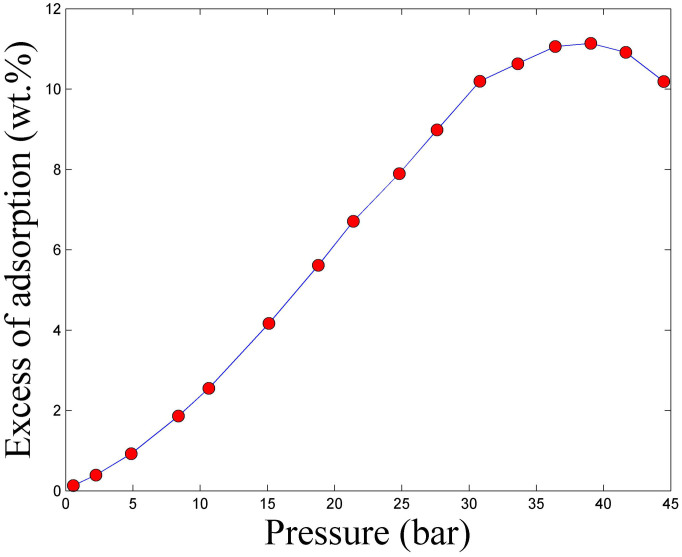
Excess CO_2_ adsorption isotherm of Li-fluorohectorite at room temperature and pressure range 0 to 45 bar. The initial part of the isotherm (0 to ca. 9 bar) represents diffusion of CO2 into the mesoporous and interlayer network^52^ of the clay powder. Experiment performed at IFE.

Figure 7 shows the excess CO_2_ adsorption isotherm of LiFh, obtained with the pcT-setup described in the Methods section. The excess adsorption is the amount of fluid taken up by the sample. The adsorption measurements were performed at room temperature and a pressure range from 1 bar up to 45 bar. The initial part of the isotherm (0 to ca. 9 bar) represents diffusion of CO_2_ into the mesoporous and interlayer network^52^ of the clay powder. Above approximately 9 bar it is likely that the swelling process of the clay has nearly finished, and this will result in increased intercalation kinetics. With further increase in CO_2_ pressure, the excess of CO_2_ is seen to rise up to around 11 wt. % at a pressure of about 38 bar. At higher pressures, the apparent amount of adsorbed CO_2_ starts to decrease, likely due to the formation of an adsorbed layer with higher density and comparable to the volume of the clay mineral, associated with approaching the critical pressure for CO_2_^53^,^54^.

## Discussion

The uptake of CO_2_ per weight of clay mineral can be inferred if it is assumed that the number of CO_2_ molecules coordinating exchangeable cations is similar to the number of H_2_O molecules within the interlayer space for the corresponding H_2_O-clay system (in the monohydrated state). This is approximately 2.4 molecules for each interlayer cation^16^,^15^. In case of LiFh and NaFh this would result in:
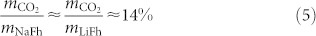
which is the same order of magnitude as measured by pcT (Figure 7), in this pressure range. This gives an amount of 3.2 mmol of CO_2_/g of LiFh. For other relevant CO_2_ capturing materials this number varies from 6.00 mmol of CO_2_/g, for e.g. metal organic frameworks (MOFs)^55^, to 5.00 mmol of CO_2_/g for Zeolites^56^. Both numbers are higher than the one we find for the clay mineral fluorohectorite. However, if we compare the adsorbed amount of CO_2_ per volume of the material, rather than per adsorbent mass, considering that the densities of zeolites (~2.2 g/cm^3^) and MOFs (~2.0 g/cm^3^) are lower than that of the clay minerals (~2.8 g/cm^3^). We find that a clay mineral, even with the cations considered here, is able to capture nearly the same mass of CO_2_ per volume (0.23 ton of CO_2_ per m^3^ of sample) as compared to the “best” zeolites (0.29 ton of CO_2_ per m^3^ of sample), or MOFs (0.32 ton of CO_2_ per m^3^ of sample). These numbers were calculated assuming 60% of packing density for all the materials. The commonly used benchmark Zeolite 13X captures 0.14 ton of CO_2_ per m^3^ of sample.

## Methods

X-ray diffraction measurements were primarily performed on an in-house (NTNU, Trondheim) Bruker NanoSTAR X-ray scattering instrument, attached to a Xenox stationary electron impact source with a copper anode, producing Kα-radiation. The scattered intensity was recorded by a two-dimensional multiwire grid Xe gas detector (HI-STAR, Bruker). The beam diameter of the setup is 400 μm and the detectable range of momentum transfer *q* is (2.5 < *q* < 7.5) nm^−1^ (*q* is defined here as *q* = 4π sin*θ*/*λ*, where *θ* is the scattering angle and *λ* the wavelength of the X-rays). The relation between *q* and *d-spacing* is *d-spacing* = 2π/*q* nm which means that the d-spacing interval is between 2.5 and 0.84 nm. Complementary X-ray scattering data were collected at the I911-4 beamline of MAX IV laboratory using a 2D CCD detector (165 mm diameter, from Marresearch, GmbH) and 0.91 Å wavelength.

The samples were mounted in a custom-made sample holder^22^ which allows temperature control in the range of −30°C to 45°C and pressures in the range from ambient to 20 bar. To allow X-rays to pass through the sample, the cell has Kapton windows on both sides of the sample volume. Internal channels connect gas from valves on the surface of the cell to the sample volume, and the gas pressure is controlled by standard reduction valves. The sample cell is depicted in Figure S3 in the supporting information.

In both X-ray setups, two-dimensional diffractograms were recorded and then azimuthally averaged to produce plots of intensity versus scattering vector, *I* vs *q*. Data reduction consisted in subtracting a background and normalizing the intensity profiles to the peak produced by the Kapton windows (see Hemmen et al.^22^ for details). The intensity, position and width of the intercalation peaks were found by fitting the peaks to Pseudo-Voigt profiles^57^,^58^.

The LiFh clay mineral used in the experiments was purchased from Corning Inc., with nominal chemical formula: *M_x_*(*Mg*_6-*x*_*Li*_x_)S*i*_8_*O*_20_*F*_4_ per unit cell^59^, where *M* is the interlayer cation (Li^+^, Na^+^ and Ni^2+^) and *x* the amount which balances the charge of clay mineral layers (*x* = 1.2 for monovalent ions, and *x* = 0.6 for divalent ions). Each sample consisted of 7 ± 1 mg of clay powder packed in the available space in the sample chamber. Typically such packed clay powder samples have a mesoporosity of about 40%^52^, which in the present case enables access of the employed gases to the layered nanoporous structures that make up the individual powder grains (here the terms mesopore and nanopore follow the IUPAC definition).

At ambient conditions (23° and 40% of relative humidity), these clay mineral samples are in the monohydrated state^9^,^60^. Since the uptake of the CO_2_ molecules may be affected by the initial H_2_O concentration^61^, we investigated dehydrated native samples. For dehydration, the samples were heated in an oven at near 150°C for more than 10 hours and in a N2 flushed atmosphere. To remove residual humidity from the cell after loading the clay, and to ensure that the sample remained dry, the cell was flushed with N_2_-gas. An X-ray scan was also recorded at ambient temperature and pressure while flushing with N_2_ to confirm that the sample remained dehydrated before starting the CO_2_ intercalation experiments. The sample was subsequently cooled to −20°C before the gas was changed to CO_2_. The gas outlet of the cell was closed and the pressure increased.

The CO_2_ used for experiments has a purity of 99.999% (Yara Praxair, grade 5). The N_2_ gas has a purity of 99.9999% (Yara Praxair, grade 6). To obtain a satisfactory signal-to-noise ratio, we varied the acquisition times from 30 to 60 minutes, depending on CO_2_ pressure, due to differences in X-ray absorption.

Pressure-composition-Temperature (pcT) isotherms were measured in a calibrated in-house built (IFE, Kjeller) volumetric Sieverts-type apparatus in order to obtain information on CO_2_ adsorption. Approximately 300 mg of Li-fluorohectorite was inserted in a sample holder and was degassed at 115°C under dynamic vacuum (<10^−6^ mbar) overnight, to remove residual humidity. Adsorption isotherms were acquired in the 0 – 45 bar range, with 3 bar step between each aliquot measurement at room temperature. The CO_2_ adsorption data were baseline corrected by the adsorption data collected from N_2_.

## Author Contributions

J.O.F. and K.D.K. planned the experiments. H.H., K.R. and L.M. designed the sample cell used for the X-ray experiments. L.M., Z.R. and G.J. da S. performed the X-ray experiments at NTNU. H.H., K.R., J.O.F. and T.P. performed the synchrotron experiments at Maxlab. P.A.S., G.N.K. and K.D.K. performed the pcT experiments and the associated data analysis. L.M., M.J. and G.J. da S. did the main part of the X-ray data analysis. J.O.F., K.D.K. and L.M. wrote the discussion of the results. L.M., H.H. and J.O.F. wrote the manuscript, and all authors revised the manuscript before submission.

## Supplementary Material

Supplementary Information Supplementary Information
