# Supplementary material for: Intercalation and Retention of Carbon Dioxide in a Smectite Clay promoted by Interlayer Cations
Source: Sci Rep. 2015 Mar 5;5:8775. doi: 10.1038/srep08775 (PMC4350078; doi:10.1038/srep08775)
Supplement: Supplementary Information [file srep08775-s1.pdf]

SUPPLEMENTARY INFORMATION

L. M (leander.michels@ntnu.no), J.O.F (jon.fossum@ntnu.no)

Intercalation and Retention of Carbon Dioxide in a Smectite Clay promoted by Interlayer Cations

L. Michels[1*], J.O. Fossum[1*], Z. Rozynek[1,a], H. Hemmen[1], K. Rustenberg[1], P.A. Sobas[2], G.N. Kalantzopoulos[2], K.D. Knudsen[2,1], M. Janek[3], T.S. Plivelic[4], G.J. da Silva[5].

[1]Department of Physics, Norwegian University of Science and Technology, NTNU. Trondheim, Norway.

[2]Physics Department, Institute for Energy Technology, IFE, Kjeller, Norway.

[3]Slovak University of Technology, Bratislava, Slovakia.

[4]MAX IV Laboratory, Lund University, Lund, Sweden.

[5]Instituto de Física, Universidade de Brasília, Brasília, Brasil.

[a]Present address: Institute of Physical Chemistry, Polish Academy of Sciences, Warsaw, Poland.

The Dehydrated Peak Dynamics

The dynamics of CO_2 intercalation can also be obtained by analyzing the dehydrated clay peak, i.e. the Bragg reflection of the clay mineral in a dry condition which has no H_2O, CO_2 or other guest molecules within the interlayer space of the clay mineral. Figure S1 shows the dynamics of the (001) dehydrated peak intensity. The dehydrated clay mineral has a d-spacing of ~ 1 nm for NiFh, NaFh and LiFh.

The intensity of the dehydrated peak decreases as CO_2 adsorbs into the interlayer space of the clay mineral. The FWHM and d-spacing of the dehydrated peak (not shown) remain nearly unchanged during the adsorption process. This is in agreement with the observations in Figure 3, which indicate that the swelling process for LiFh and NiFh are fast compared to the case of NaFh. Also the development of the d-spacing as function of time, presented in Figure S2, shows the differences in the intercalation process for these cations.

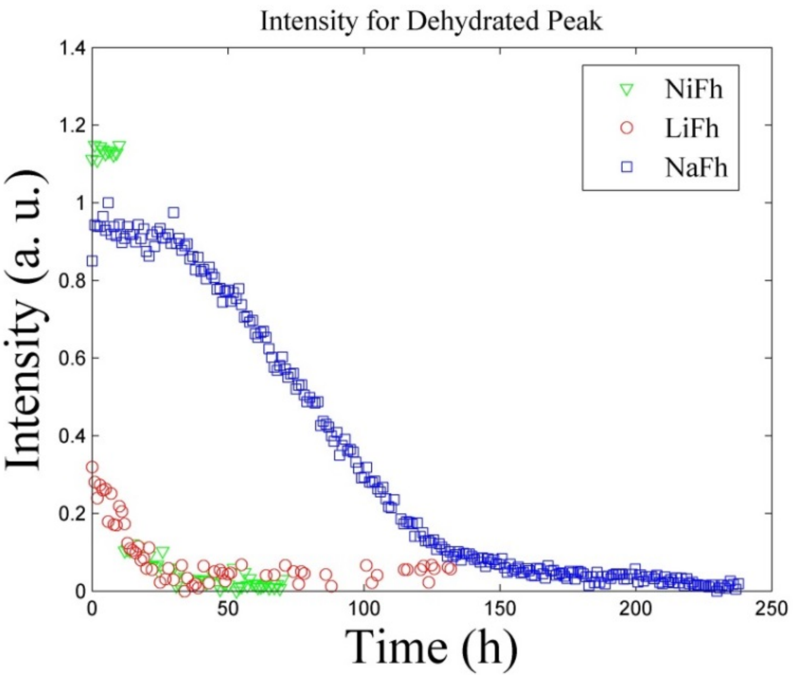

Figure S1: The intensity of the (001) dehydrated peak as function of time. As the CO_2 goes into the clay interlayer, the particles expand and the peak position appears at higher *d* values as shown in Figure 1.

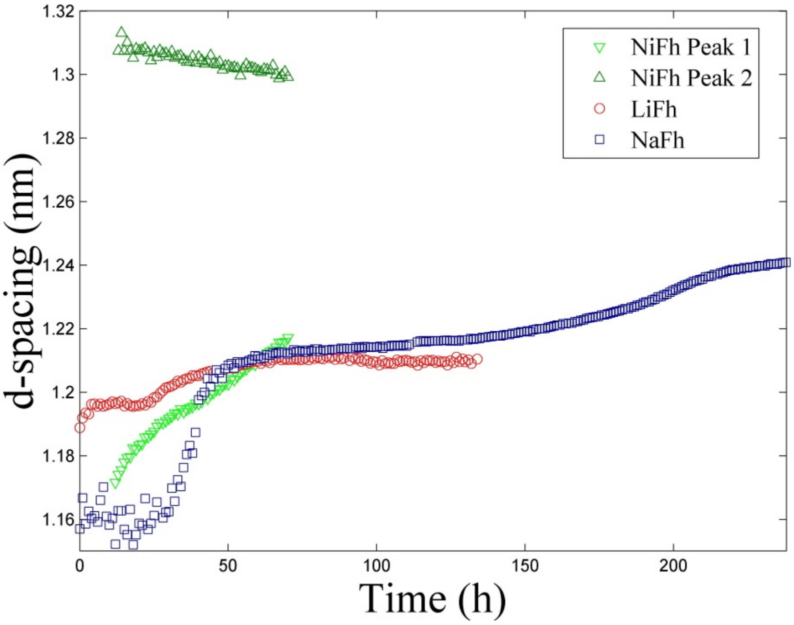

Figure S2: The d-spacing as function of time for each sample. This show the dynamics of the CO_2 intercalation peak shift.

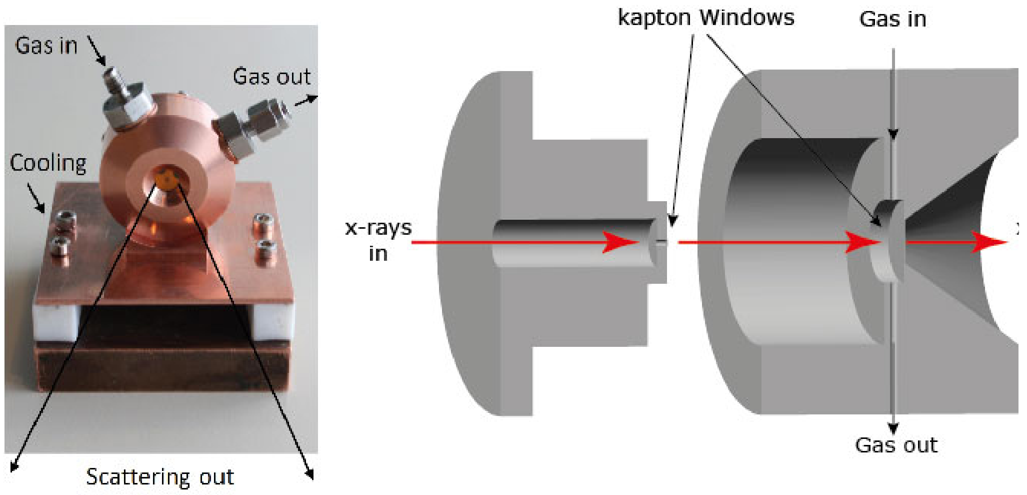

Figure S3: Picture (left) and sketch (right) of the sample cell used during the X-ray experiments. The sample volume is about 20 mm^3, with 1 mm sample thickness (i.e. X-ray path length).
